# No improvement in pregnancy and perinatal outcomes with combined luteal support in modified natural cycle frozen embryo transfer

**DOI:** 10.3389/fendo.2024.1458527

**Published:** 2025-01-15

**Authors:** Wen Zhang, Sheling Wu, Bingnan Ren, Ruolin Jia, Wenjuan Zhang, Bijun Wang, Xiaofang Du, Yichun Guan

**Affiliations:** Reproduction Center, The Third Affiliated Hospital of Zhengzhou University, Zhengzhou, Henan, China

**Keywords:** modified natural-cycle frozen-thawed embryo transfer, pregnancy outcome, luteal phase support, perinatal outcome, IVF/ICSI

## Abstract

**Objective:**

We investigated whether the addition of a luteal phase support drug benefits pregnancy and perinatal outcomes in modified natural-cycle frozen-thawed embryo transfer (mNC-FET) for women up to the age of 35 years.

**Methods:**

We analyzed the clinical data of 3658 mNC-FET cycles of women up to the age of 35 years from the Reproductive Center of the Third Affiliated Hospital of Zhengzhou University from January 2018 to December 2020 in a retrospective cohort study. The cycles were divided into three groups based on the luteal phase support protocol used. The patients in group A received a combination of progesterone soft capsules and dydrogesterone (882 cycles), those in group B received dydrogesterone only (627 cycles), and those in group C received a combination of progesterone vaginal sustained-release gel and dydrogesterone (2149 cycles). Pregnancy and perinatal outcomes were compared among the three groups.

**Results:**

Logistic regression analysis indicated that the three luteal phase support regimens were not associated with the live birth rate [OR(95% CI)B vs A=1.080, *p*=0.960; OR(95% CI)B vs C=0.252, *p*=0.291]. There were no significant differences in the newborn weight, premature delivery rate, pregnancy complications rate, and incidence of birth defects among the three groups.

**Conclusions:**

In the mNC-FET cycle, patients under the age of 35 who chose dydrogesterone alone as a luteal phase support drug exhibited no difference in the live birth rate and perinatal outcome from patients who combined dydrogesterone with progesterone soft capsules or with progesterone vaginal sustained-release gel. However, the outcome still requires confirmation by large-sample prospective studies.

## Introduction

Frozen-thawed embryo transfer (FET) cycles are widely used in the clinical setting. FET can reduce the number of ovulation-stimulating cycles in infertile patients and increase the cumulative pregnancy rate per ovulation-stimulating cycle, benefiting such patients (1). In FET, the preparation of the endometrium is critical for the success of embryo transfer. Studies have revealed that natural-cycle FET (NC-FET) and modified natural-cycle FET (mNC-FET) improve the perinatal outcome compared with the hormone replacement cycle (2); thus, NC-FET and mNC-FET, in which the timing of ovulation is triggered by human chorionic gonadotropin (hCG) (3), are more suitable for women with regular ovulation.

In assisted reproductive technology, luteal phase support is essential for the maintenance of pregnancy, and a previous Randomized controlled trial (4) revealed a significantly higher rate of live birth after administration of luteal phase support compared with non-administration in NC-FET. A single drug or a combination of drugs is commonly used as luteal phase support for the mNC-FET cycle in our center. The combined-drug protocol consists of progesterone soft capsules or progesterone vaginal sustained-release gel with dydrogesterone, and the single-drug protocol consists of dydrogesterone alone. However, it is necessary to establish whether the single drug and a combination of drugs, in addition to different routes of administration and medication frequencies, result in different pregnancy and perinatal outcomes.

Therefore, in this study we investigated differences in pregnancy and perinatal outcomes among the three luteal phase support schemes in the mNC-FET cycle to determine the most suitable protocol.

## Materials and methods

### Study design and participants

In this retrospective cohort study, we examined the clinical data of 3658 mNC-FET cycles from the Reproductive Center of the Third Affiliated Hospital of Zhengzhou University from January 2018 to December 2020. The inclusion criteria of the women were as follows: 1) age ≤35 years with a regular menstrual cycle, 2) body mass index (BMI) <28 kg/m^2^, 3) endometrial thickness ≥7 m on the hCG injection day, 4) transfer of one or two cleavage embryos or blastocysts, 5) no more than two cycles of transfer failure. The exclusion criteria of the women were as follows: 1) previously undergone preimplantation genetic testing (PGT), 2) previously accepted oocyte donation, 3) abnormal uterine environment such as intrauterine adhesions, submucosal fibroids, adenomyosis, or uterine malformations, 4) repeated implantation failure, 5) recurrent spontaneous abortion, 6) hydrops tubae profluens, 7) chromosome abnormalities. The study was approved by the ethics committee of the Third Affiliated Hospital of Zhengzhou University (2022-221-01).

### Endometrial preparation protocol

On days 10 to 12 of the menstrual cycle, follicle size and endometrial thickness were assessed using vaginal ultrasonography until the superior follicle diameter exceeded 15 mm. This measurement, combined with urinary luteinizing hormone (LH), serum LH, estradiol (E_2_), and progesterone (P) levels, was used to determine whether the follicles met the maturation criteria. When the urinary LH was positive or the serum LH level exceeded 20 IU/L, and the serum P level less than 1.5 ng/ml, a subcutaneous injection of 10,000 IU hCG (hCG, 5000 IU; Lizhu Pharmaceutical Trading Co., Ltd., Zhuhai, Guangdong, China) was administered to induce ovulation, and luteal phase support was given the following day as the intimal transformation day.

### Luteal phase support protocol

Patients in group A received progesterone soft capsules (Utrogestan; Besins Healthcare, Brussels, Belgium) at 600 mg/day and oral dydrogesterone (Abbott Biologicals, Olst, The Netherlands) at 30 mg/day, those in group B received oral dydrogesterone (Abbott Biologicals) at 30 mg/day, and those in group C received progesterone vaginal sustained-release gel (Central Pharma Ltd., Bedford, UK) at 90 mg/day and oral dydrogesterone (Abbott Biologicals) at 30 mg/day. Pregnancy was confirmed by serum hCG level on 14 days after embryo transfer, and post-pregnancy luteal phase support was continued until 30 days post-transfer. Intrauterine pregnancy was determined on 30 days after embryo transfer, the vaginal luteal support drugs was stopped on 45 days after embryo transfer, and the oral luteal phase support drug was stopped on 65 days after embryo transfer.

### Embryo transfer protocol

Cleavage-stage embryos were thawed on day4 after HCG injection, and blastocyst embryos were thawed on day6 after HCG injection. Up to two embryos were transferred. Cleavage-stage embryo scoring criteria were those of the Bourn Hall Clinic scoring system (5), with grades I–III classified as portable embryos and grades I and II classified as high-quality embryos. Blastocyst scoring criteria were those of the Gardner scoring system (5), with 4BC and above classified as portable blastocysts and 4AA, 4AB, 4BA, and 4BB classified as high-quality blastocysts.

### Data collection and outcome definition

The patient characteristics of age, duration of infertility, BMI, anti-Müllerian hormone (AMH) level, basal serum follicle stimulating hormone (FSH) level, infertility factor, endometrial thickness, number of IVF/ICSI attempts, number of previous ET cycles, number of transferred embryos, developmental stage of embryo, number of high-quality transferred embryos, pregnancy or live birth, and singletons or twins were collected from the electronic case system of our center. For patients with a gestational sac echo and singleton live birth after embryo transfer, information on pregnancy complications was retrieved during a telephone follow-up and recorded by a designated nurse at our center. Maternal and neonatal outcomes were recorded and classified according to the information provided by the patients.

The clinical outcome indicators of the three groups were observed, including hCG positive rate, clinical pregnancy rate, 12-week pregnancy rate, live birth rate, premature delivery rate, pregnancy complications rate, neonatal birth weight, and neonatal birth defects, among which the live birth rate per embryo transfer cycle was the primary outcome.

Serum hCG of 50 IU/L on 14 days after embryo transfer was defined as hCG positive. Vaginal ultrasonography on 30 days after embryo transfer was performed to confirm a clinical pregnancy (an ectopic pregnancy was also considered a clinical pregnancy), and 12-week pregnancy was defined as a clinical pregnancy that reached the 12th gestational week. Live birth was defined as the birth of a live child after 28 weeks of gestation per embryo transfer cycle. Premature delivery (PTD) was considered as a baby born before 37 weeks of gestation. For the neonatal birth weight, birth weight<2500 g was classified as low birth rate (LBW) and birth weight≥4000 g was classified as macrosomia.

### Statistical analysis

All statistical analyses were performed using SPSS version 24.0 software. Measurement data were represented using mean ± standard deviation (X ± S), and count data were represented using the rate (%).

Measurement data (age, duration of infertility, BMI, basal FSH, endometrial thickness, and number of quality embryos) were analyzed using one-way analysis of variance (ANOVA) or the Kruskal–Wallis H test according to the homogeneity of variance, and pairwise comparisons using the Bonferroni method were used. Stage of embryo at transfer, number of embryos transferred, and clinical outcomes were analyzed using the chi-square test (χ^2^). The effect of the different luteal support protocols on the live birth rate was analyzed using univariate and multivariate logistic regression models, and the odds ratio (OR) and 95% confidence interval (95% CI) were calculated. A P-value of <0.05 was considered a statistically significant difference.

## Results

A total of 3658 cycles were included in the analyses. The patients in group A (882 cycles) received a combination of progesterone soft capsules and dydrogesterone, those in group B (627 cycles) received dydrogesterone only, and those in group C (2149 cycles) received a combination of progesterone vaginal sustained-release gel and dydrogesterone.

No significant difference was found in the duration of infertility and AMH among the three groups (*P>0.05*). Significant differences in age, infertility factor, number of IVF/ICSI attempts, number of previous ET cycles, basal FSH, BMI, and endometrial thickness were observed (*P<0.05*; **Table 1**).

**Table 1:** Comparison of basic parameters among the three groups (X ± s).

Item	Group A	Group B	Group C	*F/x* ^2^	*P*-value
No. of cycles	882	627	2149		
Age (years)	30.27 ± 2.85 ^b^	29.81 ± 2.96	29.99 ± 3.11	4.654	0.010
Duration of infertility (years)	3.12 ± 2.38	2.89 ± 2.14	3.03 ± 2.25	1.838	0.159
BMI (kg/m^2^)	23.58 ± 3.16 ^b^	23.04 ± 3.18	23.32 ± 3.12	5.579	0.004
Basal FSH (IU/l)	6.87 ± 2.73 ^b^	6.23 ± 3.44	6.68 ± 2.77	9.242	<0.001
AMH (pmol/l)	24.56 ± 24.18	26.51 ± 21.52	25.39 ± 20.15	1.448	0.235
Endometrial thickness (mm)	9.74 ± 1.72 ^b^	10.10 ± 1.64	9.80 ± 1.71 ^b^	9.118	<0.001
Infertility factor				31.877	0.001
Tubal factor	58.73% (518/882)	52.95% (332/627)	55.05% (1183/2149)		
Male factor	22.68% (200/882)	25.52% (160/627)	26.99% (580/2149)		
Unexplained infertility	8.62% (76/882)	9.41% (59/627)	8.10% (174/2149)		
Others	1.25% (11/882)	3.51% (22/627)	2.75% (59/2149)		
Endometriosis	1.47% (13/882)	3.35% (21/627)	1.30% (28/2149)		
Ovulatory dysfunction	6.24% (55/882)	4.47% (28/627)	4.98% (107/2149)		
Mixed factor	1.02% (9/882)	0.80% (5/627)	0.84% (18/2149)		
Number of IVF/ICSI attempts				33.921	<0.001
1	84.24% (743/882)	93.78% (588/627)	88.69% (1906/2149)		
2	12.36% (109/882)	5.42% (34/627)	8.89% (191/2149)		
3	3.40% (30/882)	0.28% (5/627)	2.42% (52/2149)		
Number of previous ET cycles				68.791	<0.001
0	34.92% (308/882)	50.24% (315/627)	45.42% (976/2149)		
1	46.83% (413/882)	43.06% (270/627)	43.32% (931/2149)		
2	18.25% (161/882)	6.70% (42/627)	11.26% (242/2149)		

number of positives/total number in brackets. Data are presented as mean ± SD for continuous variables and % (n/N) for categorical variables. group A represents progesterone soft capsule combined with dydrogesterone;group B represents dydrogesterone; group C represents progesterone vaginal sustained-release gel combined with dydrogesteron.

b Statistically different from group B.

No significant difference was found in the ectopic pregnancy rate and the multifetal pregnancy rate among the three groups (*P>0.05*). The hCG positive rate, clinical pregnancy rate, and implantation rate in group B were all higher than those in groups A and C, with statistical significance (*P<0.001*). The 12-week pregnancy rate and live birth rate in group B were higher than those in group A (P<0.05; **Table 2**).

**Table 2:** Comparison of pregnancy outcomes among the three groups.

	Group A	Group B	Group C	*F/x* ^2^	P-value
No. of cycles	882	627	2149		
Implantation rate (%)	42.70% (556/1302)^b^	54.83% (437/797)	45.92% (1397/3042) ^b^	30.183	<0.001
hCG positive rate (%)	57.37% (506/882)^b^	67.30% (422/627)	61.05% (1312/2149) ^b^	15.313	<0.001
Clinical pregnancy rate (%)	55.67% (491/882)^b^	64.43% (404/627)	57.88% (1238/2139) ^b^	12.343	<0.001
Multifetal pregnancy rate (%)	13.85% (68/491)	9.41% (38/404)	10.58% (131/1238)	5.268	0.072
Ectopic pregnancy rate (%)	2.24% (11/491)	0.99% (4/404)	1.37% (17/1238)	2.677	0.264
12-week pregnancy rate (%)	49.55% (437/882)^b^	56.30% (353/627)	51.61% (1109/2149)	6.894	0.032
Live birth rate (%)	46.83% (413/882)^b^	53.26% (334/627)	49.19% (1057/2149)	6.124	0.047
Number of embryos transferred					
1	46.54% (215/462)	53.61% (245/457)	49.68% (624/1256)	4.628	0.099
2	47.14% (198/420)	52.35% (89/170)	48.49% (433/893)	1.319	0.517
Number of high-quality embryos transferred					
0	39.37% (124/315)	40.00% (68/170)	41.26% (302/732)	0.356	0.837
1	51.76% (206/398)	57.59% (220/382)	53.75% (566/1053)	2.805	0.246
2	49.11% (83/169)	61.33% (46/75)	51.92% (189/364)	3.162	0.206
Stage of embryo at transfer					
Cleavage	39.28% (132/336)	46.75% (79/169)	44.99% (337/749)	3.808	0.149
Blastocyst	51.47% (281/546)	55.68% (255/458)	51.43% (720/1400)	2.669	0.263

positive number/total number in brackets; Data are presented as mean ± SD for continuous variables and % (n/N) for categorical variables. group A represents progesterone soft capsule combined with dydrogesterone;group B represents dydrogesterone; group C represents progesterone vaginal sustained-release gel combined with dydrogesteron.

b represents statistically different from B.

For the perinatal outcomes of singleton and twin births, no significant differences in gestational hypertension, gestational diabetes mellitus, premature rupture of membranes, placental abruption, placenta previa, newborn weight, and premature delivery rate were found among the three groups (P>0.05; **Tables 3**, **4**).

**Table 3:** Comparison of perinatal and neonatal outcomes of singleton live births among the three groups (X ± s).

	Group A	Group B	Group C	*F/x* ^2^	P-value
Number of live births	351	307	926		
Newborn weight (g)	3413.07 ± 467.50	3413.58 ± 478.89	3425.49 ± 495.29	0.121	0.886
Low birth weight (1500–2500 g) n%	2.85% (10/351)	1.63% (5/307)	2.16% (20/926)	1.154	0.562
Very low birth weight (<1500 g) n%	0.57% (2/351)	0.33% (1/307)	0.22% (2/926)	1.446	0.487
Macrosomia (≥4000 g) n%	10.83% (38/351)	13.03% (40/307)	12.53% (116/926)	0.902	0.637
Premature delivery rate n%	4.56% (16/351)	5.54% (17/307)	4.97% (46/926)	0.333	0.847
<24 week	0	0	0	/	/
≥24 week, <28 week	0	0	0	/	/
≥28 week, <32 week	0.57% (2/351)	0.32% (1/307)	0.22% (2/926)	1.446	0.487
≥32 week, <37 week	3.99% (14/351)	5.21% (16/307)	4.75% (44/926)	0.582	0.747
Pregnancy complications rate n%	13.11% (46/351)	13.68% (42/307)	15.56% (144/926)	1.502	0.472
Gestational hypertension	2.85% (10/351)	3.58% (11/307)	3.78% (35/926)	0.649	0.723
Gestational diabetes mellitus	7.69% (27/351)	5.21% (16/307)	8.75% (81/926)	4.006	0.135
Premature rupture of membranes	1.99% (7/351)	3.91% (12/307)	2.48% (23/926)	2.568	0.277
Placental abruption	0.28% (1/351)	0.65% (2/307)	0.22% (2/926)	1.715	0.389
Placenta previa	0.28% (1/351)	0.33% (1/307)	0.43% (4/926)	0.215	1.000
Incidence of birth defects (%)	0.57% (2/351)	0.65% (2/307)	0.97% (9/926)	0.413	0.864
Limb deformity	1	0	4		
Auricular deformity	1	2	2		
Congenital heart defect	0	0	1		
Cleft lip and palate	0	0	0		
Others	0	0	2 (metabolic disease)		

Data are presented as mean ± SD for continuous variables and % (n/N) for categorical variables.

group A represents progesterone soft capsule combined with dydrogesterone;group B represents dydrogesterone; group C represents progesterone vaginal sustained-release gel combined with dydrogesteron.

**Table 4:** Comparison of perinatal and neonatal outcomes of twin live births among the three groups (X ± s).

	Group A	Group B	Group C	*F/x* ^2^	P-value
Cycles of live births	62	27	131		
Newborn weight (g)	2671.33 ± 486.08	2534.35 ± 645.67	2606.78 ± 420.98	1.708	0.182
Premature delivery rate n%	37.10% (23/62)	44.44% (12/27)	38.17% (50/131)	0.458	0.795
<24 week	0	0	0	/	/
≥24 week, <28 week	0	7.41% (2/27)	0	/	/
≥28week, <32 week	1.61% (1/62)	0	1.53% (2/131)	0.435	1.000
≥32 week, <37 week	35.48% (22/62)	37.04% (10/27)	36.64% (48/131)	0.030	0.985
Pregnancy complications rate n%	27.42% (17/62)	40.74% (11/27)	43.51% (57/131)	4.654	0.098
Hypertensive disorders of pregnancy	11.29% (7/62)	7.41% (2/27)	11.45% (15/131)	0.389	0.823
Gestational diabetes mellitus	1.61% (1/62)	3.70% (1/27)	9.92% (13/131)	4.700	0.079
Premature rupture of membranes	12.90% (8/62)	25.93% (7/27)	22.14% (29/131)	2.918	0.232
Placental abruption	0	0	0	/	/
Placenta previa	1.61% (1/62)	3.70% (1/27)	0	4.172	0.084
Incidence of birth defects (%)	0.81% (1/124)	0	0.76% (2/262)	0.427	1.000
Limb deformity	0	0	1		
Auricular deformity	0	0	0		
Congenital heart defect	1	0	1		

Data are presented as mean ± SD for continuous variables and % (n/N) for categorical variables.

group A represents progesterone soft capsule combined with dydrogesterone;group B represents dydrogesterone; group C represents progesterone vaginal sustained-release gel combined with dydrogesteron.

Univariate and multivariate regression analyses using age, BMI, basal FSH, endometrial thickness, infertility factor, number of IVF/ICSI attempts, number of previous ET cycles, and the luteal phase support drug used in the three different luteal phase support protocols were not independent factors of the live birth rate during the transfer cycle (**Table 5**).

**Table 5:** Logistic analysis of single and multiple factors influencing live birth rate.

	Single factor					Multiple factors				
	β	Wald	OR	95% CI	P-value	β	Wald	OR	95% CI	P-value
Group B	Ref.					Ref.				
Group A	-0.254	5.867	0.776	0.632–0.953	0.015	0.077	0.002	1.08	0.052–22.245	0.96
Group C	-0.164	3.235	0.849	0.711–1.015	0.072	-1.379	1.115	0.252	0.019–3.257	0.291

Univariate and multivariate regression analysis using age, BMI, basal FSH, endometrial thickness, infertility factors, number of IVF/ICSI attempts, number of previous ET cycles, and the luteal phase support protocol.group A represents progesterone soft capsule combined with dydrogesterone;group B represents dydrogesterone; group C represents progesterone vaginal sustained-release gel combined with dydrogesteron.

## Discussion

Our study revealed that in the mNC-FET cycle, women of age ≤35 years who received dydrogesterone alone as the luteal support drug exhibited no difference in the live birth rate and perinatal outcome from those who received the progesterone soft capsules or progesterone vaginal sustained-release gel combined with dydrogesterone.

Luteal phase support drugs can be administered orally, by intramuscular injection, by vaginal medication, or subcutaneously, and the effect of luteal phase support has been reported to be similar across the different routes of administration (6–8). Intramuscular injection of progesterone requires daily injection, and the long-term use of injection can cause injection-site pain, hardening of the site, reduced drug absorption, and even the formation of a sterile abscess (7, 9). In contrast, vaginal medication, which avoids the disadvantages of intramuscular injection, has a uterine first-pass effect, where the local drug concentration is maintained (10) but an increase in vaginal secretions is stimulated, causing vulvar discomfort, increased risk of vaginal infection, and the possibility of sexual intercourse affecting drug absorption (11). Oral progesterone has low bioavailability (12) and may have adverse effects such as drowsiness. Dydrogesterone is a reverse-transcribed progesterone, is a more selective progesterone receptor agonist than progesterone, and has low affinity for androgens and glucocorticoid receptors (13), and its oral administration can avoid the inconvenience and side effects of vaginal medication or intramuscular injection (12, 14, 15).

The maintenance of pregnancy cannot be separated from normal luteal function. The role of luteal phase support in IVF fresh embryo transfer is widely recognized (7). FET in the hormone replacement cycle has itself does not induce luteal generation and is completely dependent on exogenous progesterone to maintain luteal function (16). It remains controversial whether luteal phase support is required for FET (4, 17–19). Previous studies suggested that the corpus luteum, produced by spontaneous ovulation, can maintain embryo implantation (20). Although luteal insufficiency may lead to implantation failure and abortion (21), a previous report revealed that the incidence of luteal insufficiency was 3.7–20% in infertile patients. Even in the normal ovulation of primary or secondary infertility, about 8.1% of patients exhibited luteal insufficiency (22), and progesterone regulated the immune mechanism to reduce the abortion rate (23). A systematic review and meta-analysis in 2021 of 15 studies involving 416 reports (24) suggested that luteal phase support using progesterone was significantly associated with a higher clinical pregnancy rate and live birth rate in NC-FET and mNC-FET. Furthermore, the LH level at the use of hCG to trigger, which may have affected endometrial events and the clinical pregnancy rate (25). In addition, luteal phase support may be able to correct synchrony at transfer. Therefore, we preferred to use progesterone for luteal phase support in mNC-FET.

There are no uniform criteria for luteal phase support in mNC-FET. Jin et al. used a combination of progesterone vaginal sustained-release gel and dydrogesterone as the luteal phase support in NC-FET (26), Shi et al. (27) and Hu et al. (28) used dydrogesterone alone as the luteal phase support in mNC-FET, and Peeraer et al. (29) used progesterone soft capsules in mNC-FET. Previous studies have indicated that dydrogesterone and vaginal drugs do not work well if used alone, and their combination should be used (30). If progesterone alone or vaginal luteal phase support alone has shown little effectiveness, their combined use may be not avoided (31), and the combination of vaginal medication and intramuscular administration exhibited a better clinical outcome than the use of each approach alone (32). However, studies were performed on FET in the hormone replacement cycle, and studies may have been related to the absence of auto-luteal formation of HRT-FET. Such absence increases the need for greater luteal phase support compared with the case of mNC-FET. Some investigators believed that the combination of luteal phase support drugs through the vagina and other routes is not supported by evidence, assuming that the clinician decided to combine different drugs the drug to exclude the possibility of unsatisfactory drug administration (8).

In our study, the luteal phase support in mNC-FET used dydrogesterone alone or in combination. Upon increasing luteal phase support drugs, the live birth rate did not improve, although it increased the cost and discomfort borne by the patient. A randomized, single-center, parallel controlled trial exhibited similar sustained pregnancy rates for mNC-FET with dydrogesterone and with vaginal sustained-release gel. In the same study, vaginal irritation, vaginal discharge, and interference of sexual intercourse with drug absorption were lower with oral medication than with vaginal medication (33). A systematic review and meta-analysis on luteal phase support protocol (34) explored fresh embryo transfer and frozen embryo transfer in mNC-FET, as well as the clinical outcome achieved using dydrogesterone, which was similar to that using progesterone soft capsules. These results were consistent with our study, in which satisfactory clinical outcomes were achieved with dydrogesterone alone.

The development of the placenta has a direct impact on perinatal outcomes (35) from the establishment of fetoplacental circulation at 3 weeks after fertilization until the complete formation of placental function at 12 weeks of pregnancy, during which progesterone improves the uterine environment and promotes the establishment of placental function (36). During normal placental development, estrogen and progesterone are critical, and altered sex steroid hormone levels may contribute to placenta-related complications (37–39). In early pregnancy, low progesterone levels may lead to placenta accreta (40), and high progesterone in early third trimester has been associated with the later development of pre-eclampsia (41). In our study, no difference was found in the premature delivery rate, newborn weight, gestational diabetes mellitus, gestational hypertension, premature rupture of membranes, placental abruption, placenta previa, and incidence of birth defects. The effect of a second luteal phase support drug on the perinatal outcome was not obvious in mNC-FET, but because the perinatal outcome is also influenced by many factors, such as both parental characteristics, ART treatment characteristics, after FET, maternal tubal factor, ovulatory dysfunction, and unexplained infertility (42, 43), so the impact of different luteal phase support protocols on the perinatal outcome requires further investigation.

This study had the following limitations. 1) The study was retrospective with some bias; therefore, additional prospective studies are required to validate our results. 2) Because maternal complications and offspring outcomes were obtained by telephone conversation and reported by patients, the data were incomplete. 3) Luteal phase support drugs were voluntarily selected by patients: most of the patients in the mNC-FET cycles did not choose to be treated with dydrogesterone alone, and the majority chose progesterone vaginal sustained-release gel and oral dydrogesterone, resulting in differences in basic indicators between the groups and in the sample size. Especially in the dydrogesterone group, high proportion of blastocyst transfer cycles, and a high proportion of high-quality embryos, these indicators will improve the pregnancy outcomes. Because of this, the live birth rate was studied as the dependent variable, and univariate and multivariate regression analyses using age, BMI, basal FSH, endometrial thickness, infertility factors, number of IVF/ICSI attempts, number of previous ET cycles, and luteal phase support drugs in different luteal phase support schemes did not independently influence the live birth rate.

## Conclusion

Patients with age ≤ 35 years who chose dydrogesterone alone as the luteal phase support drug in mNC-FET cycles exhibited clinically effective and safe maternal and infant outcomes. However, this finding requires further confirmation by large-sample prospective studies.

## Data Availability

The raw data supporting the conclusions of this article will be made available by the authors, without undue reservation.
